# Epigenetic Regulation of Hyaluronan-Associated Genes in the Brain: Identifying Key Regulatory Sites

**DOI:** 10.3390/epigenomes10020028

**Published:** 2026-05-01

**Authors:** Rosalyn E. Acevedo, Esther Walton, Karen R. Mifsud

**Affiliations:** 1 Bristol Veterinary School, University of Bristol, Langford Campus, Bristol BS40 5DU, UK; kb22472@bristol.ac.uk; 2 Department of Psychology, University of Bath, Bath BA2 7AY, UK; e.walton@bath.ac.uk

**Keywords:** hyaluronan, epigenetics, extracellular matrix, brain

## Abstract

Hyaluronan (HA) is a ubiquitous extracellular matrix (ECM) component that is gaining significant attention for its diverse roles in cell signalling and disease. The biological functions of HA are dependent on its molecular weight (M_w_): low M_w_ polysaccharide chains drive stimulatory processes such as inflammation and angiogenesis, whereas high M_w_ HA is stabilising and anti-inflammatory. Growing evidence indicates that HA is integral to brain function. The composition of HA in the brain is regulated by the balance of enzymatic synthesis and degradation, mediated by different isoforms of hyaluronan synthase (HAS) and hyaluronidase (HYAL) respectively. Fluctuating expression of the genes encoding the HAS and HYAL enzymes has been implicated in neuropathology and ageing, with some studies providing evidence towards epigenetic regulation of these genes. The regulatory environment of the brain confers a unique balance of enhanced protection alongside the requirement for maximum flexibility. This scoping review focuses on summarising current knowledge regarding epigenetic regulation of HAS and HYAL genes in neural contexts, as well as identifying gaps in knowledge against which future research can be directed. Understanding how these genes are regulated, particularly through epigenetic mechanisms, provides insight into how HA is regulated in the brain, facilitating understanding regarding its function in brain health and disease.

## 1. Introduction

Following its identification in 1934 [1], hyaluronan (HA) was long considered an inert, structural component of the extracellular matrix (ECM), providing a hydrated framework in which cells are embedded. The discovery of HA-binding proteins, also known as hyaladherins [2,3,4], revealed the ever-increasing roles for HA in a myriad of cellular processes, including inflammation [5], joint development and function [6,7], wound healing [8], and cancer progression [9]. More recently, HA has also been implicated in brain function and disease, with roles in neuroplasticity [10,11], inflammation [12] and neurogenesis [13]. Understanding how HA synthesis and degradation are regulated at the epigenetic level may provide insight into how HA production is fine-tuned to meet the demands of different tissues, while also identifying potential therapeutic targets in disease where dysregulated HA metabolism contributes to pathogenesis.

### 1.1. Hyaluronan Biochemistry, Distribution and Functionality in the Brain

HA is a non-sulphated glycosaminoglycan (GAG) consisting of repeating disaccharide units of N-glucuronic acid and N-acetylglucosamine. HA chains can reach above 8000 kDa in mammals; however, the size distribution is dependent on the species [14]. HA is synthesised at the cell membrane by hyaluronan synthase (HAS) enzymes, membrane-bound glycosyltransferases that catalyse the condensation reaction between uridine diphosphate-glucuronic acid and uridine diphosphate-N-acetyl-D-glucosamine to form the HA polymer [15]. Mammals have three synthase genes, *Has1*, *Has2* and *Has3.* Interestingly, each HAS enzyme produces distinct sizes of HA, with HAS2 synthesising the highest molecular-weight (M_w_) HA chains, followed by HAS1 and HAS3 [16]. HA is degraded through multiple mechanisms, including enzymatic digestion by hyaluronidases (HYAL) [17], oxidative fragmentation by reactive oxygen species (ROS) [18], and mechanical shear forces [19].

HA is distributed throughout the brain as part of various structures; in the diffuse interstitial matrix [20], attached to membrane-bound proteins of the endothelial glycocalyx at the blood-brain barrier (BBB) [21], and/or in ‘unique lattice-like structures’ surrounding specific neurons known as perineuronal nets (PNNs) [22]. The content of HA in various brain regions changes throughout development [13,23] and ageing [24], as well as in response to microglia depletion [25], injury [26] and disease [27].

HA has been ascribed functional roles in a number of biological processes including neurogenesis, neural plasticity, neuroinflammation, and the formation and maintenance of PNNs [10,28,29]. Adult neurogenesis in the subgranular zone (SGZ) of the hippocampus is critical for hippocampal-dependent learning and memory [30]. High M_w_ HA, signalling via CD44, significantly reduced proliferation of primary neural stem cells (NSCs) collected from the hippocampus of mice compared with controls [13]. Furthermore, stereotactic injection of hyaluronidase, to degrade HA in the hippocampus of mice, significantly increased proliferation of NSC compared with vehicle-injected controls, an effect abolished in CD44 knockout mice [13]. These results suggest a key regulatory role for HA in hippocampal function, mediated by CD44 in SGZ neurogenesis.

Hyaluronidase-mediated degradation of high M_w_ HA in the rat hippocampus in vivo attenuated spatial behavioural deficits induced by repeated blast exposure to improve working memory [31]. Additionally, HA degradation enhanced N-methyl-D-aspartate (NMDA)-dependent long-term potentiation (LTP) in murine embryonic stem cell cultures in vitro [32]. In contrast, hyaluronidase treatment reduced L-Type voltage-dependent calcium channel-dependent signalling in murine hippocampal slices, impairing LTP induced by five theta trains. Notably, this deficit was rescued by subsequent incubation of the treated slices with HA [11]. Bilateral injection of hyaluronidase was shown to inhibit contextual fear conditioning in mice [11]. Collectively, these findings indicate that hyaluronidase and hyaluronan bioactivity may play an important role in modulating neural plasticity and cognitive function. In addition to the effects of HA degradation, studies have also investigated the role of HA synthesis in neural plasticity and cognition. Inhibiting HAS with chronic 4-methyumbeliferone (4-MU) treatment improved long-term memory retention; however, these improvements reverted back to baseline levels following the cessation of treatment [33]. These studies indicate that a tightly regulated balance between HA synthesis and degradation may be crucial for maintaining optimal neural plasticity and cognitive function.

A role for HA in neuroinflammation has also been implicated. Hyaluronidase injection into mouse hippocampal Cornu Ammonis 1 (CA1) induced an acute upregulation in the expression of inflammatory genes including toll-like receptor 2 (TLR2) and chemokine (C-C motif) ligand (CCL) 2, 3, and 5 [34]. Furthermore, high M_w_ HA decreased lipopolysaccharide-mediated inflammatory cascades in rat primary microglial cultures [12]. These findings support the current consensus that HA-mediated inflammatory responses are dependent on molecular weight [35], and indicate that this relationship is maintained within brain-specific contexts, where high M_w_ HA confers anti-inflammatory properties whilst smaller HA fragments are pro-inflammatory.

PNNs are stabilising and protective structures surrounding neurons in the brain and spinal cord [10]. These vital structures act to restrict synaptic plasticity following the formation of neural circuits [36]. HA appears to be integral to the formation of PNNs; expression of HAS3 was critical to induce the formation of a PNN-like structure in an embryonic kidney cell line, in conjunction with cartilage link protein-1 (CRTL1) and aggrecan, but neither was sufficient alone [37]. There is strong evidence for the expression of both *HAS2* and *HAS3* mRNA and related proteins in neurons surrounded by PNNs [38,39], but limited evidence that HAS1 protein may also contribute to PNNs [39]. Since each isoform synthesises HA of different lengths [40], this raises the possibility that HA chains of differing M_w_ contribute to the PNN structure. Furthermore, regional differences in HA composition of PNN in mouse brains have been reported [41]. Hyaluronidase treatment reduced the number of PNN-expressing neurons in primary mouse hippocampal cultures and was associated with a significant increase in epileptiform activity [42]. Whilst intriguing, further research is required to make a causal association between these features.

### 1.2. Hyaluronan Regulation in the Brain

A number of processes have been implicated in the regulation of HA in physiology and disease. These can be divided into those acting directly on existing HA to alter its molecular weight (i.e., mechanical forces, enzymes, ROS), or more commonly, processes acting on the expression or activity of HA-associated enzymes (cell signalling pathways, inflammatory cytokines, growth factors and metabolic state), to alter the balance of HA synthesis-HA degradation and thus impact the HA environment [43,44]. Interestingly, HA itself has been reported to regulate its own expression, through feedback on HAS expression in various cell types in a molecular weight-dependent manner [45,46]. Post-translational modifications (phosphorylation, ubiquitination, glycosylation) have also been reported to regulate the expression of HA-related genes [47].

Despite HA being ubiquitously distributed throughout the body, its concentration and M_w_ vary markedly between regions [48]. The ECM of the brain is compositionally distinct from that of most peripheral tissues. This difference contributes to the brain’s specialised structure and function and creates a unique regulatory environment. Notable differences in brain ECM compared to that of the periphery include relatively high levels of GAGs, including HA, and the prominence of lecticans—proteoglycans containing a lectin domain such as neurocan, brevican, versican and aggrecan [49]. In addition, unlike peripheral tissues, the brain ECM is not characterised by the extensive fibrous protein network that provides structural support to tissues in other areas of the periphery. This difference likely contributes to the brain’s soft and highly dynamic mechanical environment [50].

Furthermore, the BBB establishes a highly specialised microenvironment that differs substantially from that in peripheral tissues, with distinct immune, metabolic, oxidative and stress-related regulation [51]. The selectively permeable BBB restricts entry of hormones, immune mediators and metabolites, buffering neural tissue from the systemic changes and exposures that influence regulatory mechanisms in peripheral organs [51].

**Figure 1:**
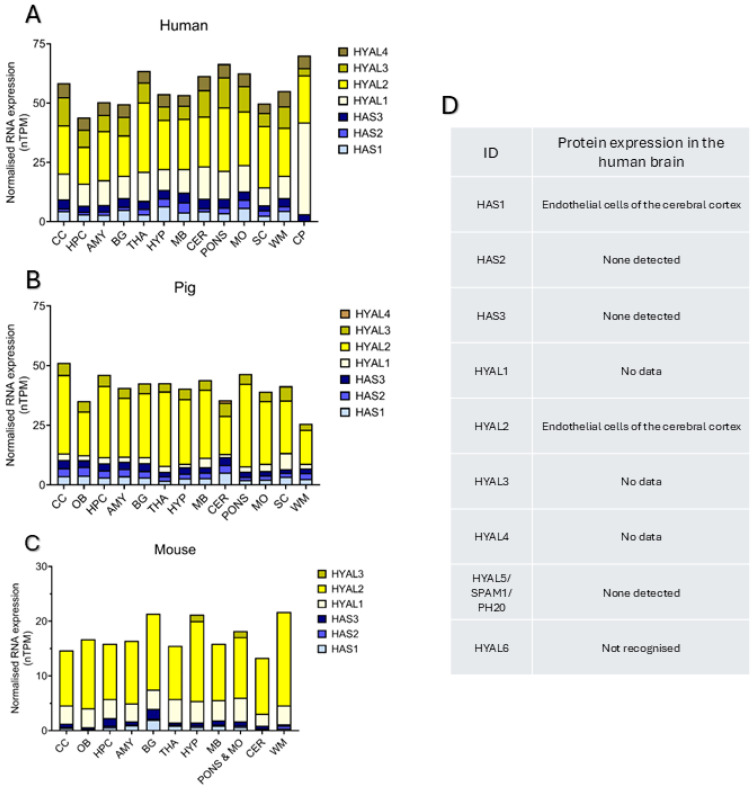
HA-related gene and protein expression across species. Expression of hyaluronan synthase (HAS) and hyaluronidase (HYAL) isoform RNA in human (**A**), pig (**B**) and mouse (**C**) brains by region and expressed as normalised transcripts per million (nTPM) according to the Human Protein Atlas (HPA) database Version: 25.0, updated 11 November 2025, accessed 29 January 2026 [54]. Abbreviations: cerebral cortex (CC), olfactory bulb (OB), hippocampal formation (HPC), amygdala (AMY), basal ganglia (BG), thalamus (THA), hypothalamus (HYP), midbrain (MB), cerebellum (CER), medulla oblongata (MO), spinal cord (SC), white matter (WM), choroid plexus (CP). (**D**) Characterisation of protein expression of HA-related enzymes in human brain samples from HPA confirmed the expression of HAS1 and HYAL2; however, expression of HAS2 and HAS3 was not detected by this resource, despite evidence of brain expression from the literature. Expression of HYAL1, HYAL3 and HYAL4 was not explored as part of the HPA project so no data on the expression of these enzymes in the HPA database exists.

Given the tissue specificity of the ECM composition, regulatory mechanisms controlling HA metabolism could also be brain-specific. In addition to the three mammalian hyaluronan synthases (HAS1–3), there are six mammalian hyaluronidases (HYAL1–4, SPAM1 aka HYAL5, and HYAL6), although HYAL6 is not expressed in humans and only found in the testes of mice [52]. Of these, all but SPAM1 showed significant expression of associated RNA in the brain according to the Human Protein Atlas (HPA, Figure 1A–C) [53,54], whereas protein expression was less robust (Figure 1D). This is likely due to limitations of the HPA (effectiveness of antibodies/tissue characteristics) as opposed to lack of expression, given that other studies have provided evidence for expression of HAS1–3 and HYAL1–2 at the protein level in neural cells [55,56]. Epigenetic modifications are one way by which individual characteristics, exposures and a dynamic cellular environment can influence gene expression without altering the underlying DNA sequence [57].

### 1.3. Epigenetic Regulation and Its Relevance for HA

Chemical modification of specific nucleotides within the DNA sequence can alter gene transcription, resulting in changes to RNA expression without altering the underlying DNA sequence [57]. The most common DNA modification is methylation of cytosine residues located in CpG islands (regions of DNA expressing high levels of cytosine–phosphate–guanine (CpG) dinucleotides) [58]. In the nucleus, DNA is packaged around histone proteins to form chromatin, which can itself be densely packaged as heterochromatin, impeding DNA replication and transcription, or lightly packaged euchromatin, facilitating gene transcription [59]. The extent to which DNA is condensed can be determined by covalent modification of histone tails, generally taking the form of acetylation, methylation, phosphorylation, ubiquitination and O-GlcNAcylation [60]. MicroRNA (miRNA) are also emerging as important contributors to the epigenetic regulation of genes, either as targets of DNA/histone modifications or because they are themselves capable of controlling epigenetic effectors (e.g., methyltransferases/deacetylases), therefore influencing the expression of genes [61]. The role of miRNAs in HA metabolism is summarised in a review by Vigetti et al. [47]. The majority of these studies reported, however, focussed on cellular environments outside those of the brain.

The degree to which epigenetic marks in non-neural contexts can be used to predict those occurring in the brain is limited [62,63]. Even the most comprehensive analysis reports that only a small proportion of CpG sites show correlated methylation patterns between blood and brain, with current correlative estimates of between 8 and 20% [63,64]. These findings indicate that epigenetic data derived from peripheral tissues may not accurately predict epigenetic regulation within the brain. Most adult neurons are post-mitotic and divide infrequently [65]. Epigenetic modifications occurring in the brain are therefore relatively stable, allowing molecular changes to accumulate across the lifespan and persistently influence neuron function until cell death. Furthermore, as infrequently dividing cells, neurons have been reported to demonstrate higher prevalence of DNA methylation (DNAm) occurring outside traditional CpG sites [66]. As a result, neurons especially are exposed to a distinct profile of signalling molecules that shape DNAm patterns, histone modifications and chromatin accessibility [67,68]. Therefore, epigenetic regulation of hyaluronan-related genes in the brain should be characterised independently and may not conform to inferences drawn from the periphery.

For these reasons, this review aims to identify epigenetic modifications of genes implicated in HA metabolism specifically within a neural context in mammals, focusing on *HAS1–3*, *HYAL1–4* and *SPAM1*. For the purposes of this review, our definition of epigenetic modifications includes DNAm, histone modification and regulation by miRNAs. Understanding this body of literature will enable more targeted studies to ascribe functional roles for epigenetic regulation of HA in the mammalian brain and identify knowledge gaps, which can be the focus of future studies.

## 2. Results

**Figure 2:**
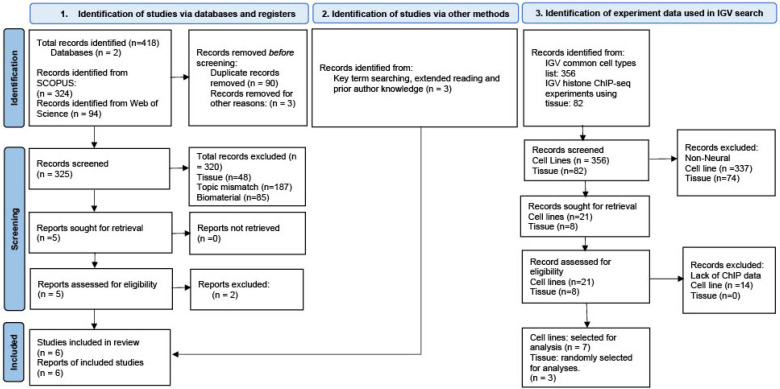
PRISMA flow chart outlining search methodology. Publications identified from systematic search databases and publications identified from prior author reading are represented under columns 1 and 2, respectively. Experimental data collected from chromatin immunoprecipitation (ChIP)-sequencing (ChIP-seq) experiments in both neural cell lines and human tissue is summarised under column 3. A summary of all identified and selected articles as well as the cell lines and tissue used in ChIP-seq data analysis can be found in Supplementary Tables S1 and S2 respectively.

Despite search strategies identifying 324 and 94 manuscripts from Scopus and Web of Science databases respectively (Section 4, Figure 2), manual review of these hits identified three manuscripts of relevance based on inclusion/exclusion criteria (Section 4, Supplementary Table S1). Three additional manuscripts were identified as meeting inclusion criteria based on the authors’ prior knowledge. These six studies investigated DNAm and miRNA as epigenetic mechanisms targeting *HAS1–3*, *HYAL1–4*, and *SPAM1* genes and are discussed in detail below.

### 2.1. Epigenetic Regulation of HYAL3 in Attention-Deficit Hyperactivity Disorder

Neurogenesis and synaptic plasticity are essential for normal brain development during early life [69]. Studies of neurodevelopmental disorders have identified alterations in the expression of genes associated with these processes, including *HAS* and *HYAL* [70]. There is also evidence that epigenetic mechanisms may contribute to this dysregulation. One study has been identified from this evidence, which specifically investigated the potential epigenetic regulation of *HAS* or *HYAL* genes in attention-deficit hyperactivity disorder (ADHD), and relevant findings were extracted.

Wang et al. [71] implemented a comprehensive multi-omics framework to investigate the potential role of gene transcription, alternative splicing (AS) and DNAm in attention-deficit hyperactivity disorder (ADHD). The study integrated ADHD genome-wide association study (GWAS) summary statistics with multiple quantitative trait loci (QTL) datasets, including expression QTL (eQTL), splicing QTL (sQTL) and methylation QTL (mQTL) [71]. Using this integrative approach, *HYAL3* was identified as a significant associated gene in 13 of 14 brain tissue types (all except putamen), supporting its role as a potential brain-wide gene related to ADHD. Both alternative splicing and gene expression were implicated as mechanisms by which *HYAL3* could potentially contribute towards ADHD development, identified through both a splicing transcriptome-wide association study (sTWAS) and expression transcriptome-wide association study (eTWAS). DNAm was proposed as a potential mechanism regulating the expression of *HYAL3*. Five DNAm sites within a +/−2 mega base window of *HYAL3* were identified as potentially relevant by target probes: cg0365530, cg22973319, cg02490920, cg16913124 and cg06980053 [71]. Given that *HYAL3* was consistently detected as an associated gene for ADHD across nearly all brain regions, it is probable that a shared regulatory mechanism across brain tissues, such as DNAm-mediated transcriptional changes to *HYAL3* expression, may contribute to ADHD.

### 2.2. Epigenetic Regulation of HAS and HYAL in Neurodegenerative Disorders

It is widely agreed that DNAm is altered in genes associated with Alzheimer’s disease (AD) such as amyloid beta precursor protein (*APP*), presenilin 1 (*PSEN1*), and microtubule-associated protein tau (*MAPT*) [72]. Such changes may contribute to the onset, progression and pathology of AD. A recent investigation has provided evidence for hypomethylation (loss of methyl groups from DNA) of *HAS2* in the hippocampus of AD patient post-mortem (PM) tissue. In a study of genome-wide DNAm within the hippocampus, PM samples from AD patients exhibited marked hypomethylation of the CpG site at position 122,466,955 on chromosome 8 (cg06688910) compared to age-adjusted control samples [73]. Though this site is ~180 kilobases (kb) downstream of the *HAS2* transcription start site (TSS), and typically out of range for a regulatory region, Genomic Regions Enrichment of Annotations Tool (GREAT) analysis [74], which allows for extended regulatory domains up to 1-megabase to capture long-range regulatory effects, indicated an association with this DNAm site and *HAS2* in this study. Additional studies will be needed to confirm if any functional relationship exists.

Likewise, a meta-analysis of data generated from prefrontal cortex samples from over 1000 AD patients collected as part of four cohort studies identified consistent DNAm changes associated with the Braak stage (progression level) of AD [75]. To account for genomic inflation, the researchers applied Bacon correction, and identified 2767 high-confidence, differentially methylated CpGs that achieved significance at the 5% false discovery rate. Within this high-confidence dataset, four specific CpGs were identified as being associated with *HAS3* and *HYAL2* [75]. Cg07855319, located in an ‘open sea’ region ~18 kb upstream of *HAS3*, was consistently hypomethylated across all four cohorts. CpG sites cg13341668 and cg05118960 in the 5′ untranslated region (UTR)/TSS1500 region of *HYAL2* were found to be hypermethylated (addition of methyl groups to DNA) across cohorts [75]. Hypermethylation of these CpG sites has been associated with reduced adjacent gene expression [76]. Finally, cg15275312 was hypomethylated across cohorts; however, it is unclear whether this locus is associated with *HYAL2* or *TUSC2* as GREAT and Illumina annotations provide conflicting regulatory associations [75].

Changes in DNAm at HA-associated genes have been investigated in a mouse model of demyelinating disease. Experimental autoimmune encephalomyelitis (EAE) is a mouse model of multiple sclerosis (MS) [77]. MS is a degenerative and demyelinating disease that has been linked to epigenetic changes in the central nervous system (CNS) [78]. Induction of the EAE model in female mice was accompanied by changes in brain DNAm levels [77]. Long-read nanopore sequencing identified 490 differentially methylated genes (DMGs) in the brains of mice with EAE compared to controls, including *Has1*, which was hypermethylated [77]. Hypermethylated *Has1* was proposed to be one of many genes associated with aberrant immune cell responses in EAE. Specifically, *Has1* activity was associated with pro-inflammation, T-cell activation antigen presentation, dendritic cell maturation and phagocytosis [77].

### 2.3. Epigenetic Regulation of HAS and HYAL Across Ageing

In mammals, brain HA levels are reported to undergo significant changes across the lifespan. During early development and the postnatal period, HA levels rise rapidly, reaching a peak within the first postnatal week [79]. This increase is thought to be associated with heightened levels of synaptic plasticity during neurodevelopment [43,80]. As the brain matures into adulthood, HA levels decline and remain relatively stable [79]. In older age, HA increases again, with studies showing regional accumulation of HA as well as the degradation of high M_w_ HA into more abundant, soluble low-*M_w_* HA [24,81]. These shifts in the HA environment across the lifespan may be driven by developmental and age-related changes in epigenetic regulation of *HAS* or *HYAL* genes. Genomic DNA, extracted from newborn and adult mice, underwent DNAm analysis, revealing methylation of a specific region of DNA in the 5′UTR segment of *Hyal2* in adult mice that was not methylated in DNA extracted from newborns, thus implicating developmental regulation of *Hyal2* by DNAm [82]. This corresponds to earlier findings that Hyal2 is expressed at the protein level in newborn mice brains, but expression is absent in adulthood [83].

Another study investigated the age-related regulation of *Has2* by the *let-7* miRNA family within the rat retina and vitreous [84]. The *let-7* miRNA has the potential to be regulated by epigenetic modification [85], and as such, could represent a mechanism for epigenetic regulation of HA in neural tissues due to high expression in human neural retinas [86]. This study measured the expression of seven *let-7* isoforms (*let-7a-i*) across three life stages: newborn (1–3 days), young adult (2 months) and aged (12 months). In the newborn retinas, which contain neural cells, *Has2* expression peaked, while *let-7* levels were at their lowest [84]. Rats in the young adult group had significantly lower levels of *Has2* compared to newborn rats. In parallel, specific *let-7* isoforms were upregulated in the young adult group and maintained stable, high levels into the aged group [84]. Since the 3′UTR region of *Has2* is a predicted target of *let-7* miRNAs [87], the inverse correlation between *let-7* and *Has2* indicates that *let-7* may play a regulatory role in the inhibition of *Has2* expression. This interaction was validated using a luciferase reporter assay, which confirmed that *let-7* targets the 3′UTR of the *HAS2* mRNA within human Müller cells (a specialist type of glial cell) [84]. These findings provide a clear mechanism by which the synthesis of HA can involve miRNA regulation in neural tissues.

### 2.4. Key Regulatory Sites for Histone Modifications Within HAS/HYAL Genes

**Figure 3:**
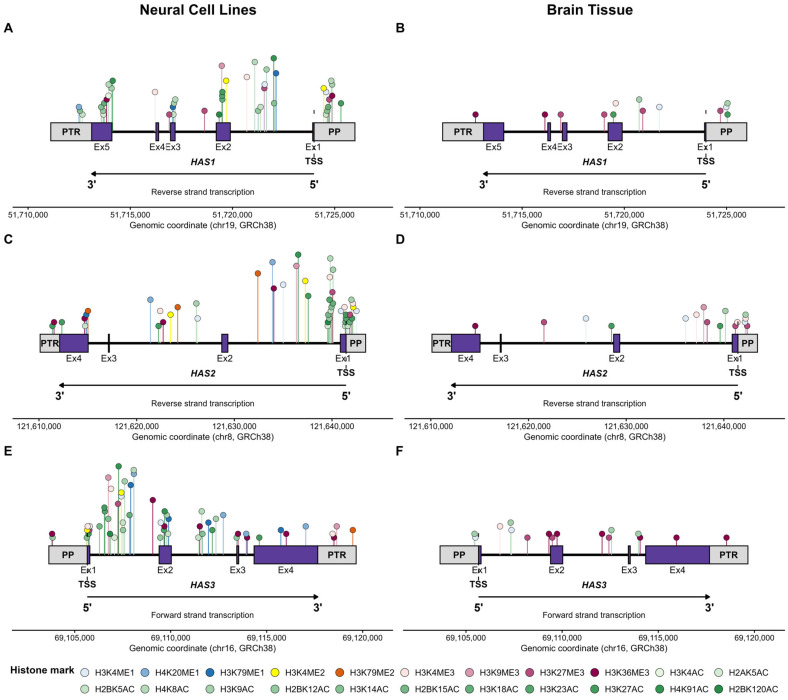
Distribution of histone modifications across hyaluronan synthase (HAS1–3) genomic regions in neural cell lines (**A**,**C**,**E**) and brain tissue (**B**,**D**,**F**). One location of each type of histone mark is displayed per region. This location was calculated as an average location across all examples of marks present in either cell lines or tissue. Regions are defined as intron, exon, proximal promoter (PP; 2000 bp upstream of transcription start site, TSS), and post-transcriptional region (PTR; 2000 bp downstream of the transcription end site). Histone marks are colour coded by modification type: methylation marks (ME1, ME2, ME3) are shown in blue, yellow–orange and pink–purple shades respectively, and acetylation marks (AC) are shown in shades of green. Where multiple marks of the same type are present, lighter shades indicate lower lysine residue numbers. The height of the lollipop sticks does not convey any relevant biological information, but rather is for visualisation purposes. Raw data is available in Supplementary Table S2. This figure was constructed in R version 4.5.2.

**Figure 4:**
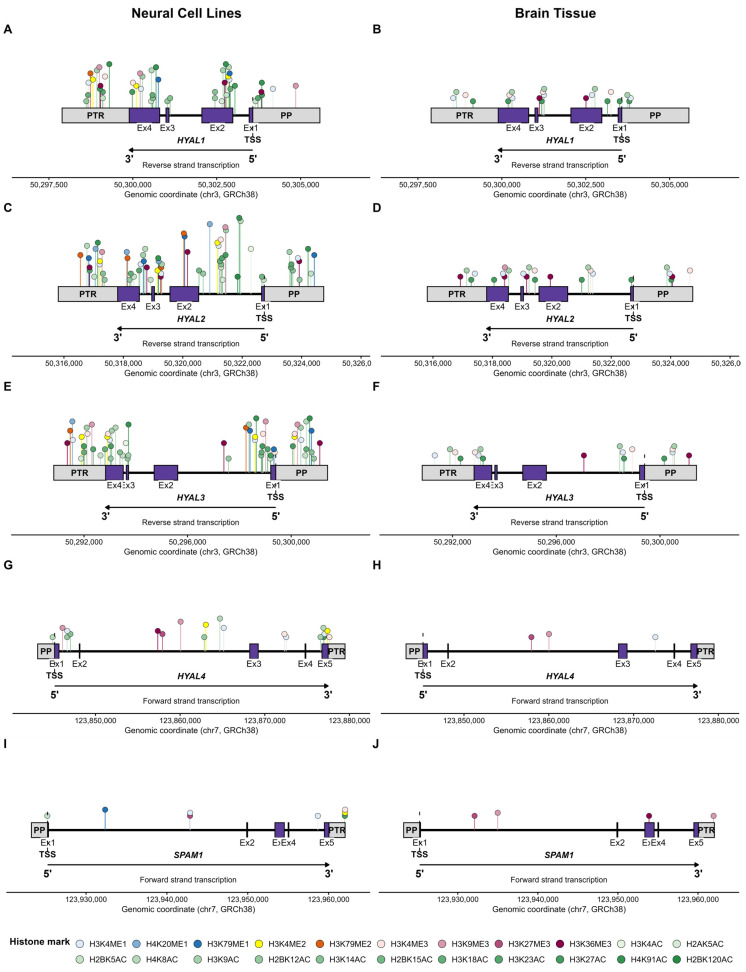
Distribution of histone modifications across hyaluronidase (HYAL1–4, SPAM1) genomic regions in neural cell lines (**A**,**C**,**E**,**G**,**I**) and brain tissue (**B**,**D**,**F**,**H**,**J**). One location of each type of histone mark is displayed per region. This location was calculated as an average location across all examples of marks present in cell lines or tissue. Regions are defined as intron, exon, proximal promoter (PP; 2000 bp upstream of transcription start site, TSS), and post-transcriptional region (PTR; 2000 bp downstream of the transcription end site). Histone marks are colour coded by modification type: methylation marks (ME1, ME2, ME3) are shown in blue, yellow–orange and pink–purple shades respectively, and acetylation marks (AC) are shown in shades of green. Where multiple marks for the same type are present, lighter shades indicate lower lysine residue numbers. The height of the lollipop sticks does not convey any relevant biological information, but rather is for visualisation purposes. Raw data is available in Supplementary Table S2. This figure was constructed in R version 4.5.2.

Given the limited number of studies identified through our literature search, and the lack of studies examining the specific role of histone modifications for HA-related gene expression, we targeted the Encyclopaedia of DNA Elements (ENCODE) database to assess evidence for histone modifications associated with HA-related genes of interest (*HAS1–3*, *HYAL1–4*, *SPAM1*) [88]. We focused on the most common human cell lines from a neural lineage [89], on the assumption that these are likely to contain the widest range of modifications (Section 4). In addition, datasets derived from human brain tissue were also probed to maximise translational relevance. Using ENCODE datasets generated from neural cell lines and brain tissue, we identified potential sites of epigenetic regulation through histone modification by summarising the datasets uploaded as part of the ENCODE project for hyaluronan synthases (Figure 3) and hyaluronidases (Figure 4).

**Figure 5:**
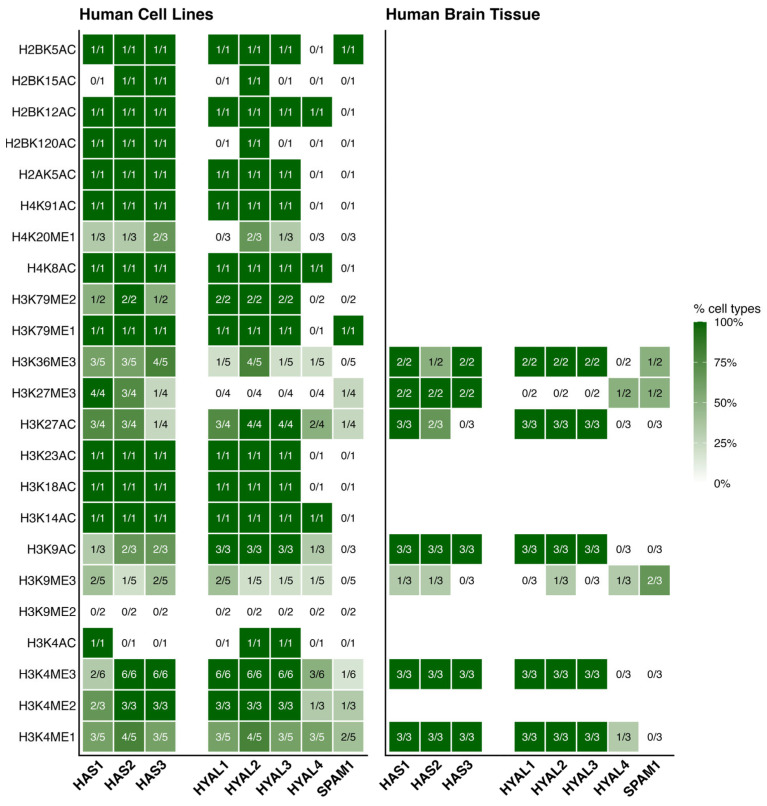
The presence of histone modifications at HAS and HYAL gene loci across human cell lines and brain tissue. This heatmap shows the proportion of cell lines and tissue types with histone target peaks detected within +/−2 kb of the transcription start site (TSS) and transcription end site (TES). Values in each cell indicate the number of cell/tissue types with a peak for that mark within the genomic region over the total number of cell types examined. In total, 9 human cell lines were examined and 3 human tissue samples. Peaks were identified from selected datasets from the ENCODE database. Raw data available in Supplementary Table S2. This figure was constructed in R version 4.5.2.

A greater number of histone marks were identified in the human cell line datasets compared to the brain tissue datasets (Figure 5). All histone marks showed presence in at least one cell type in at least one gene, except H3K9me2, which did not show peaks in any cell line at any of the genes investigated (Figure 5). *HAS2*, *HAS3* and *HYAL2* exhibited the broadest range of histone modifications present, each with 21/22 marks in at least one of the samples investigated (Table 1). *SPAM1* showed the narrowest range of histone mark presence, and the lowest number of total peaks observed. Whilst the data are not directly comparable, a low number of potential regulatory sites for *SPAM1* is not surprising since this enzyme is predominantly expressed in the testis (Human Protein Atlas, www.proteinatlas.org). *HAS3* had the greatest number of total peaks observed (Table 1).

## 3. Discussion

Whilst literature directly assessing a functional role for epigenetic regulation of HA-related genes in a neural context is generally lacking, this review did highlight studies in which DNAm in CpG sites within HA-related genes, and expression of miRNAs targeting HA-related genes, are correlated with specific pathological states [71,73,75,77]. Furthermore, utilising ChIP-seq datasets of the ENCODE project uncovered extensive regions across all HA-related genes that were robustly enriched for specific histone modifications. This raises the possibility that these sites could have functional relevance for HA dynamics in the brain by controlling the transcription of HA-related genes through epigenetic mechanisms.

### 3.1. Potential Impact of Epigenetic Regulation of HA Dynamics

DNAm of HA-related genes associated with specific neurological disorders were stable across multiple brain regions [71] and different cohorts [75], indicating that DNAm may induce long-term changes in HA homeostasis by altering the balance of HA synthesis and degradation through regulation of *HAS* and *HYAL* expression respectively. This maybe especially true for neurons, in which limited cell division would sustain long-term alterations. Neural cell-type specificity of DNAm changes was not investigated as part of the studies reported here, but offers an exciting avenue for future research.

MicroRNA activity on the other hand can be rapidly induced in a region-specific manner to bring about dynamic changes to gene transcription [90]. Whilst this rapid regulation was not apparent in the study of retina cells, miRNAs were implicated as potential regulators of HA-related gene expression across the lifespan [84]. It will be interesting to discover the temporal dynamics of HA-related gene regulation by microRNAs in future studies.

Whilst we did not discover any experimental papers investigating regulation of HA-related genes by histone modifications through our literature searches, we did discover evidence that histone modifications are prevalent across genomic regions encoding HA-related genes in neural-derived samples (Figure 5). Generally, there appears to be a broader regulation of HAS genes by a wider range of modification compared with HYAL genes, although this difference has not been confirmed by statistical analysis. This broader regulation could be because HAS is essential for HA synthesis. HAS isoforms may be subjected to greater fine-tuning processes via epigenetic regulation to ensure that the resultant HA environment contains the correct molecular weight distribution to meet the functional requirements of a specific region. HYALs, on the other hand, act on pre-existing HA. Therefore, regulatory processes of these genes at the epigenetic level could be less complex. One caveat against this theory is that in the Golgi/endoplasmic reticulum (ER) of some mammalian cells (rabbit kidney epithelial cells/rat keratinocytes), a pool of HAS proteins exist [91]. These proteins are then trafficked to the plasma membrane in response to additional demands for HA synthesis [91]. It is unclear if this type of HAS protein pool exists in cells of neural origin. If it did, it would imply that isoform recruitment to the plasma membrane is controlled (or not) at the level of trafficking, not at the levels of transcription/translation. Therefore, the importance attributed to the epigenetic regulation of HAS/HYAL genes in the control of hyaluronan metabolism could be expanded to focus on post-transcriptional factors important in the trafficking of HAS proteins to the plasma membrane.

Whilst the evidence presented supports regulation of HA-related genes by all types of epigenetic modifications, it is unlikely that these marks impact gene expression in isolation. *HYAL1* is not expressed in bladder cancer cells due to DNAm of specific cytosines in the promoter region of *HYAL1* gene [92]. Treatment with a DNA demethylating agent, 5-AzaC, induced *HYAL1* expression in these cell lines, confirming a causal epigenetic regulation of *HYAL1* by DNAm. Interestingly, this induction was enhanced further in the presence of Trichostatin A (TSA), a histone deacetylase inhibitor, implicating an associated role for histone acetylation in the expression of *HYAL1*. TSA treatment alone, however, could not induce *HYAL1* in these cell lines, indicating that histone acetylation required DNA demethylation for its effects of *HYAL1* translation [92]. It is therefore likely that epigenetic processes work in concert to fine-tune the expression of HA-related genes.

### 3.2. Limitations and Recommendations

Whilst our search returned a good number of articles, many were excluded at initial review due to lack of relevance, resulting in three relevant papers. Three additional papers with a focus on epigenetic regulation of HA-related genes were discussed based on the authors’ knowledge of the relevant literature. Whilst this strategy introduces potential selection bias, the limited number of relevant studies justifies their inclusion. It is likely that more articles containing data within scope exist but were not identified by our search. For example, investigations that study the impact of epigenomic changes on gene expression generally do so at the whole-genome level. Individual genes may not be specifically mentioned but only found by searching the Supplementary Materials. Alternatively, older papers may use different terminology or gene nomenclature, therefore avoiding capture.

Papers identified through our literature search had some limitations, and therefore conclusions should be drawn with caution. For example, in Altuna et al. [73], the investigation of DNAm within AD PM brains used control brains, which were on average ~30 years younger [73]. Although analyses were age-adjusted, it is unknown how well the adjustment corrected for potential age-related differences in methylation. Nevertheless, the study by Altuna et al. [73] remains useful for the purpose of this review since it identifies sites where *HAS* and *HYAL* genes were epigenetically regulated.

Exploring the ENCODE dataset allowed us to identify histone marks occurring in the vicinity of HA-related genes. Our investigations were restricted to common cell lines and tissue samples. Whilst this procedure provided a systematic approach to data selection, an extended analysis may have revealed a greater number of relevant histone modifications.

## 4. Materials and Methods

### 4.1. Search Strategy

Although this work functions as a scoping review, it incorporates principles of systematic methodology to reduce bias and ensure a comprehensive identification of the relevant literature. Initially, we conducted two advanced literature searches in the Scopus database [93], which was extended to the Web of Science database to broaden the scope (Figure 2). All publications found to be relevant to previously defined inclusion/exclusion criteria (see section “Selection Criteria”) were selected for greater review.

An initial Scopus search conducted on 10 October 2025 found 287 results, in which the following search terms were used:

(TITLE-ABS-KEY (hyaluron*) AND TITLE-ABS-KEY (“brain” OR “neur*” OR “glia*” OR “astrocytes” OR “microglia*” OR “brain organoids” OR “neural stem cells” OR “cortical”) AND TITLE-ABS-KEY (“epigenetic” OR “histone” OR “DNA methylation” OR “microRNA”))

The abstracts of these 287 results were screened against selection criteria (see Section 4.2), and each result was categorised into potentially relevant or not. A reason for each exclusion was given (see Supplementary Table S1). Of the 287 publications identified, three were excluded prior to screening: one article had been retracted by the publishing journal and two were graphical abstracts rather than full articles (see Supplementary Table S1). Next, 284 studies were then screened for relevance to our investigation into the epigenetic regulation of hyaluronan-associated genes (Figure 2). Of the remaining 284 publications, 158 were classified as ‘topic mismatch’ if they did not investigate or mention the pre-defined epigenetic processes at HAS/HYAL genes, 39 did not investigate epigenetic marks in normal (non-cancerous) brain tissue, and 82 used HA as a biomaterial. This resulted in four full-text articles assessed for eligibility in a second screening. Following this, a further two were excluded due to no explicit mention of specific sites of epigenetic regulation in either HAS or HYAL genes in neural tissue. Finally, two publications were relevant for deeper analysis [77,84].

Following the initial search, a second Scopus search was conducted with adapted search terms, with the aim of identifying additional relevant papers. These terms were more specific, including the HAS and HYAL genes of interest.

((TITLE (hyaluron* OR “HAS1” OR “HAS2” OR “HAS3” OR “HYAL*”) OR ABS (hyaluron* OR “HAS1” OR “HAS2” OR “HAS3” OR “HYAL*”) OR AUTHKEY (hyaluron* OR “HAS1” OR “HAS2” OR “HAS3” OR “HYAL*”)) AND (TITLE (“brain” OR “neur*” OR “glia*” OR “astrocytes” OR “microglia*” OR “brain organoids” OR “neural stem cells” OR “cortical”) OR ABS (“brain” OR “neur*” OR “glia*” OR “astrocytes” OR “microglia*” OR “brain organoids” OR “neural stem cells” OR “cortical”) OR AUTHKEY (“brain” OR “neur*” OR “glia*” OR “astrocytes” OR “microglia*” OR “brain organoids” OR “neural stem cells” OR “cortical”)) AND (TITLE (“epigenetic” OR “histone” OR “DNA methylation” OR “microRNA”) OR ABS (“epigenetic” OR “histone” OR “DNA methylation” OR “microRNA”) OR AUTHKEY (“epigenetic” OR “histone” OR “DNA methylation” OR “microRNA”)))

This search generated 37 results. Duplicates were removed, and then the same exclusion methods and categorisations used in the previous search were implemented. This resulted in one additional article [71] for deeper review (Figure 2) [71]. To ensure a comprehensive search and minimise database-specific bias, the above two queries were also conducted in the Web of Science core collection database [94]. However, this search did not generate any further new, relevant publications (Figure 2). The adapted queries were as follows:

Search 1: TS = (hyaluron* AND (“brain” OR “neur*” OR “glia*” OR “astrocytes” OR “microglia*” OR “brain organoids” OR “neural stem cells” OR “cortical”) AND (“epigenetic” OR “histone” OR “DNA methylation” OR “microRNA”))

Search 2: TS = ((hyaluron* OR HAS1 OR HAS2 OR HAS3 OR HYAL*)

AND (“brain” OR neur* OR glia* OR astrocytes OR microglia* OR “brain organoids” OR “neural stem cells” OR cortical)

Due to the limited number of publications identified by our literature searches, we included an additional three relevant papers in the main review that were identified through extended reading [73,75,82]. These papers did not come up in either Scopus search due to the absence of ‘hyaluronan’, ‘HAS’, ‘HYAL’, and ‘DNA methylation’ in the main body of either paper. Rather, data on epigenetic regulation of *HAS* and *HYAL* genes was reported in the associated supplementary data for these publications.

### 4.2. Selection Criteria

Studies were included if they identified and provided evidence for the existence of regulatory, epigenetic sites on the endogenous gene of interest: *HAS1–3*, *HYAL1–4*, and *SPAM1*. Experimental evidence supporting the proposal that the existence of these sites had to come from a mammalian source.

Studies were excluded if the mechanism of genome regulation explored did not fit within the confines of our ‘epigenetics’ definition. ‘Epigenetics’ was defined as the following: ‘Changes in gene expression that occur without the alteration of a DNA sequence, through the mechanisms of DNA methylation, histone modification or microRNA’. For example, studies on regulations via long non-coding RNA, or post-transcriptional regulations, were excluded. Additionally, studies in which the biological material was genetically modified or cancerous were excluded, to ensure relevance and translatability of the results.

### 4.3. Interactive Genome Viewer Search

Given that the literature search failed to identify any articles focused on the regulation of HA-related genes by histone modifications, we manually probed publicly available data through the ENCODE database using the Integrative Genome Viewer (IGV) [95,96].

Analysis was restricted to central nervous system-derived cell lines and human brain tissue to ensure relevance to our aim of identifying sites of epigenetic regulation at HAS/HYAL genes within the brain. Neural cell lines were selected from the ENCODE list of most common cell types (2007–2012; see details below) [89], as these cell lines have the most extensive publicly available ChIP-seq datasets [95,97].

Our aim with this analysis was to identify regions of HA-related genes with the potential for regulation by histone modifications, without making any assumptions about the functional relevance of such modifications. As such, we wanted to maximise the number of potential sites identified. The previous literature has indicated that cancer cell lines can display specific patterns of histone regulation that are distinct from comparable healthy cell lines [98]. As such, cancer-derived cell lines included in the common cell type list were included in this part of the analysis, given their potential to maximise the breadth of histone modifications identified that have potential to modify HA-related genes in neural contexts.

The common cell lines selected included one cell line derived from embryonic stem cells and differentiated to neurons (H1-neurons), two astrocyte cell lines (NH-A and HAc) and four cancerous cell lines (SK-N-SH, SK-N-SH + retinoic acid, BE2_C, SK-N-MC). In addition, datasets from three human brain tissue ChIP-seq experiments were screened to increase biological and translational relevance of our findings and to enable a comparison of histone modification patterns between post-mortem brain tissue and common in vitro cell lines [89]. Tissues selected include the hippocampus (HPC), temporal lobe (TL) and dorsolateral prefrontal cortex (dlPFC). A complete list of cell lines and tissues included in this study as well as the histone marks targeted is provided in Supplementary Table S2. Utilisation of PEAK files only ensured that the sites identified from the database correlated to regions in which histone modifications were statistically enriched above background (input/control DNA) levels. Where available, replicated peak datasets were used, as these represented consensus signals observed across biological replicates and therefore reduce false positives. For datasets in which replicated peaks were not available, the most representative peak dataset was used.

Data was aggregated manually by reviewing each dataset for our selected cell types individually in the IGV. For each dataset, peak tracks were visualised and the genomic regions corresponding to the HAS and HYAL gene loci were inspected. Peaks located within +/−2 kb of the TSS and the TES were identified and relevant information recorded. Presence or absence of peaks as well as peak statistics within these regions were recorded in a summary table (Supplementary Table S2).

## 5. Conclusions

Studies discussed above provide evidence that genes essential for HA synthesis and degradation can be regulated at the epigenetic level by DNA methylation, a wide variety of histone modifications and via microRNA activity. The additional layer of regulation that epigenetic modification provides is likely to facilitate fine-tuning of the HA environment, promoting co-ordinated neural responses for ongoing neurogenesis, neuronal plasticity and neuroimmune function. Key sites within HA-related genes that offer potential for epigenetic regulation in both human and rodent tissue, as well as human-derived cell lines, were identified. Focusing on these sites could enable more focused future studies investigating the possibility for epigenetic regulation of HA-related genes, and therefore elucidate the regulatory mechanisms underlying the functional roles ascribed to hyaluronan in brain physiology and pathology. Furthermore, this review highlights a pressing need for additional research to establish the causal mechanistic relationships between epigenetic modifications and HA bioactivity in neural contexts.

## Figures and Tables

**Table 1:** Summary values for each gene across all datasets. The number of marks indicates the total number of distinct histone modifications that show at least one enriched peak associated with a given gene identified in any of the cell types or tissues investigated. The total number of histone modifications with enriched peaks was 22. Total peak number indicates the total number of individual enriched peaks associated with a given gene across all histone marks and cell types investigated. The mean signal value refers to the average ChIP-seq signal intensity across all peaks associated with a given gene, pooled across all histone marks and cell types.

Gene	Number of Marks Present (/22)	Total Peak Number	Mean Signal Value
HAS1	20	148	4.87
HAS2	21	227	6.76
HAS3	21	257	7.51
HYAL1	17	180	9.56
HYAL2	21	270	8.47
HYAL3	19	229	17.58
HYAL4	11	28	4.33
SPAM1	9	18	3.59

## Data Availability

No new data were created or analysed in this study. Data sharing is not applicable to this article.

## Supplementary Materials

The following supporting information can be downloaded at: https://www.mdpi.com/article/10.3390/epigenomes10020028/s1; Table S1: Literature search results; Table S2: Histone modifications of HA-related genes extracted from the ENCODE database.

## Abbreviations

The following abbreviations are used in this manuscript:

4-MU	4-methyumbeliferone
AC	Acetylation
AD	Alzheimer’s disease
ADHD	Attention-deficit hyperactivity disorder
APP	Amyloid beta precursor protein
AS	Alternative splicing
BBB	Blood–brain barrier
CA1	Cornu ammonis 1
CCL	Chemokine (C-C motif) ligand 2
CNS	Central nervous system
ChIP	Chromatin immunoprecipitation
ChIP-seq	Chromatin immunoprecipitation-sequencing
CpG	Cytosine–phosphate–guanine
CTRL1	Cartilage link protein 1
dlPFC	Dorsolateral prefrontal cortex
DNA	Deoxyribonucleic acid
DMG	Differentially methylated gene
DNAm	DNA methylation
ENCODE	Encyclopaedia of DNA Elements
EAE	Experimental autoimmune encephalomyelitis
ECM	Extracellular matrix
ER	Endoplasmic Reticulum
eQTL	Expression QTL
eTWAS	Expression transcriptome-wide association study
GWAS	Genome-wide association study
GREAT	Genomic Regions Enrichment of Annotations Tool
GAG	Glycosaminoglycan
HA	Hyaluronan
HAS	Hyaluronan synthase
HPA	Human Protein Atlas
HPC	Hippocampus
HYAL	Hyaluronidase
IGV	Integrative Genome Viewer
KB	Kilobase
LTP	Long-term potentiation
MAPT	Microtubule-associated protein tau
ME	Methylation
mQTL	Methylation QTL
miRNA	microRNA
M_w_	Molecular weight
MS	Multiple sclerosis
NSC	Neural stem cell
NMDA	N-methyl-D-aspartate
PNN	Perineuronal net
PM	Post-mortem
PP	Proximal promoter
PSEN1	Presenilin 1
PTR	Post-transcriptional region
QTL	Quantitative trait loci
ROS	Reactive oxygen species
RNA	Ribonucleic acid
SGZ	Subgranular zone
SPAM1	Sperm adhesion molecule 1, aka HYAL5
sQTL	Splicing QTL
sTWAS	Splicing transcriptome-wide association study
TES	Transcriptional end site
TL	Temporal lobe
TLR2	Toll-like receptor 2
TSA	Trichostatin A
TSS	Transcriptional start site
UTR	Untranslated region
